# Development and validation of the Mentalizing Emotions Questionnaire: A self-report measure for mentalizing emotions of the self and other

**DOI:** 10.1371/journal.pone.0300984

**Published:** 2024-05-06

**Authors:** Lea A. Kasper, Sophie Hauschild, Anna Berning, Julia Holl, Svenja Taubner

**Affiliations:** 1 Psychological Institute, University Heidelberg, Heidelberg, Germany; 2 Institute for Psychosocial Prevention, University Hospital Heidelberg, Heidelberg, Germany; Medical University of Vienna, AUSTRIA

## Abstract

Mentalizing describes the ability to imagine mental states underlying behavior. Furthermore, mentalizing allows one to identify, reflect on, and make sense of one’s emotional state as well as to communicate one’s emotions to oneself and others. In existing self-report measures, the process of mentalizing emotions in oneself and others was not captured. Therefore, the Mentalizing Emotions Questionnaire (MEQ; current version in German) was developed. In Study 1 (*N* = 510), we explored the factor structure of the MEQ with an Exploratory Factor Analysis. The factor analysis identified one principal (R^2^ = .65) and three subfactors: the overall factor was *mentalizing emotions*, the three subdimensions were *self*, *communicating* and *other*. In Study 2 (*N* = 509), we tested and confirmed the factor structure of the 16-items MEQ in a Confirmatory Factor Analysis (CFI = .959, RMSEA = .078, SRMR = .04) and evaluated its psychometric properties, which showed excellent internal consistency (α = .92 - .95) and good validity. The MEQ is a valid and reliable instrument which assesses the ability to mentalize emotions provides incremental validity to related constructs such as empathy that goes beyond other mentalization questionnaires.

## Introduction

Mentalizing describes the capacity to perceive and understand oneself and others in terms of mental states (emotions, beliefs, thoughts, and desires) [1]. Mentalization is closely linked to emotion regulation and its development, whereby mirroring and the resulting co-regulation of emotional states by the caregiver have a central function in the self-regulation of one’s own emotions [2, 3]. Fundamental to the development of mentalizing in early childhood is building a self-representation by a caregiver’s mirroring of the child’s primary emotional states [1]. The mirroring gives meaning to the inner sensations of the child that are unconscious, intrapsychic embodied experiences. The child learns about their emotional states by internalizing the caregiver’s feedback (visual, vocal, and body-related) as mental representations [1, 4]. This enables the child to gain knowledge of their emotions. Accordingly, mentalization of emotions can be divided into three components (1) identifying, being aware of, as well as naming emotions, (2) processing emotions in the sense of developing an understanding and (3) communicating emotions to others. In the development of mentalizing in childhood, after having gained access to one’s own emotions via bio-social-feedback (mirroring), the child learns to ascribe (identify and process) mental states to others, e.g. of the caregiver as a part of a goal-corrected partnership—which is well documented by the Theory-of-Mind body of research [5, 6].

As mentalization becomes challenging when emotions intensify, learning to better mentalize emotions is considered a key mechanism of change in psychotherapy [7]. Mentalizing one’s own emotions and the emotions of others enables appropriate coping with external and internal stressors, the regulation of emotions, and the establishment of stable interpersonal relationships [1]. Especially in psychotherapy, it is important to facilitate mentalizing emotions more effectively to initiate change [8, 9].

Jurist [10, 11] proposed mentalized affectivity as the most mature form of emotion regulation: it is separated into three aspects of emotion regulation, (1) identifying emotions in the context of individual circumstances, personal memories as well as exploring the source of emotions, (2) processing in the sense of modulation and regulation of emotions (e.g. emotions can be changed in duration and intensity) and (3) expressing emotions conceptualized as communication of one’s own emotions internally as well as externally to others. It is important to note that there are differences in the conceptualization of identifying, processing, and expressing/communicating in relation to emotion regulation according to Jurist [11] and mentalizing emotions operationalized in this study. According to Jurist [10, 11] identifying is seen as part of mentalizing emotions, whereas processing and expressing emotions to the self in his concept is closely linked to emotion regulation. Communicating emotions to others is an additional functional interpersonal competence as stated by Arbeitskreis OPD-3 [12]. Jurist [11] and Greenberg [13] strongly emphasized the importance of mentalization in the process of emotion regulation: prior to, during, and after the refining and modulating of the emotion. This is precisely why it is so important to take a more in-depth analysis of the individual components involved in mentalizing emotions. Overall, it should be pointed out that the theory of mentalized affectivity [11] mainly focuses on emotion regulation in the self, excluding understanding emotions of others.

Mentalizing is a complex construct and therefore difficult to measure [14, 15]. It includes both a self-reflective and an interpersonal component, whereas including mentalizing others sets it apart from self-reflection [1, 16]. The gold standard for capturing mentalizing is the Reflective-Functioning Scale (RF Scale) [16], which can be applied to interviews such as the Adult-Attachment Interview [17] or therapy transcripts [18]. Coding with the RF Scale is based on a comprehensive manual that highlights aspects of mentalizing mental states such as openness, awareness of the nature of mental states, development aspects, and reflecting on current emotions while interpreting others [1, 16]. Conducting and transcribing interviews or therapy transcripts for the use of the RF Scale is time-consuming and reliability requires extensive training.

For a more economical assessment of mentalization, a variety of different questionnaires have been developed [14]. The most often used questionnaires for the investigation of mentalization in Germany seem to be the Reflective Functioning Questionnaire (RFQ-6) [19], the Mentalization Questionnaire (MZQ) [20] and the Certainty about Mental States Questionnaire (CAMSQ) [21], although these have not yet been validated with the RF Scale.

The RFQ-6 [19] focuses on cognition and non-mentalized emotions, e.g. describing limited communication due to emotions ("When I get angry I say things without really knowing why I am saying them."). The MZQ [20] assesses maladaptive characteristics of mentalizing, in which non-mentalizing of emotions is included. Here, individual items refer to delayed identifying of own emotions (e.g. "Sometimes I only become aware of my feelings in retrospect.") as well as failed processing (e.g. "Often I don’t even know what is happening inside of me.") and failed communicating (e.g. "Talking about feelings would mean that they become more and more powerful."). Within the MZQ the emphasis on the inability to mentalize emotions is noteworthy as well as the neglect of mentalizing emotions as a comprehensive process. The CAMSQ [21] refers to the self as well as to others and thus includes an important point of mentalization theory [1]. Six of the 20 items can be associated with mentalizing emotions in a broader sense, e.g. processing emotions of the self ("I understand my feelings.") or identifying emotions of others ("I can tell when a person in a group is feeling awkward."). In addition to emotions, the CAMSQ refers to thoughts and motives; it does not explicitly refer to identifying, processing, and communicating mentalized emotions.

As in present mentalizing questionnaires no subscale is dedicated to mentalizing emotions, hence this important facet of the mentalization construct is not assessed. Furthermore, only the CAMSQ distinguished between self and others.

Phenomena such as emotional blindness [22], also known as alexithymia, psychological symptoms [23] and level of personality functioning [24] as well as intelligence [25] are related to mentalization. Therefore, when developing questionnaires to assess mentalization, these constructs and their associated questionnaires should also be taken into account, e.g. Short Alexithymia Scale (SAS-3) [26], Symptom-Checklist K9 (SCL-K-9) [27], Level of Personality Functioning Scale–Brief Form 2.0 (LPFS-BF) [28] and Berlin Test for the Assessment of Fluid and Crystallized Intelligence—Short Form Crystallized Intelligence (BEFKI GC-K) [29].

Questionnaires outside the core mentalization construct capture specific components of mentalizing emotions or concepts closely associated with it: for instance measures assessing epistemic trust (Epistemic Trust, Mistrust and credulity Questionnaire, ETMCQ) [30], attributional complexity (Attributional Complexity Scale, ACS) [31], beliefs about emotions (Emotion Belief Questionnaire, EBQ) [32], emotion knowing and understanding (GEMOK-Blends) [33], emotion regulation (Emotion Regulation Questionnaire, ERQ) [34], empathy (Empathy Quotient, EQ) [35] and mentalized affectivity (Brief Mentalized Affectivity Scale, B-MAS) [13]. With the exception of attributional complexity and empathy, these questionnaires do not consider the understanding of others and thus miss an essential aspect of mentalizing.

The ETMCQ [30] differentiates between epistemic trust, mistrust, and credulity. Epistemic trust is defined as openness to the reception of social knowledge that is regarded as personally relevant and of generalizable significance [36]. It has a close conceptual relationship to mentalization, which is also developed in early attachment experiences. However, the ETMCQ, despite the conceptual proximity, does not assess identifying, processing or communicating mentalized emotions (“Sometimes, having a conversation with people who have known me for a long time helps me develop new perspectives about myself.”). The ACS [31] measures the attributional complexity describing the degree to which a more complex explanation for human behavior is chosen. The ACS focuses on the processing component in relation to the self and others („I have thought a lot about the family background and personal history of people who are close to me, in order to understand why they are the sort of people they are.”). However, the focus here is on behavior, attitudes, and beliefs, leaving emotions out of the equation. The EBQ [32] differentiates between the perceived general controllability and usefulness of positive and negative emotions. This construct taps into processing of emotions ("It doesn’t matter how hard people try, they cannot change their negative emotions."). What is noticeable about the EBQ is that it does not refer directly to the self or others, but instead refers to people in general. In the GEMOK-Blends emotion knowing and understand in relation to others is tested. In contrast to mentalization, where hypotheses are made about the mental states of others, the GEMOK-Blends refers to the correct or incorrect attribution of emotions in others (“Which of the following emotions describe best what Daniel was experiencing during this episode?”). Thereby, it includes parts of the identifying and processing component. The ERQ [34] distinguishes between two common emotion regulation strategies: suppression and reappraisal. Suppression could be assigned to communicating emotions ("I keep my emotions to myself.") and reappraisal in a broader way to processing emotions ("When I want to feel more positive emotion, I change the way I’m thinking about the situation."). In the EQ [35], only a few items relate directly to mentalizing emotions. Items such as "It is hard for me to see why some things upset people so much." can be linked to problems in processing emotions of others. The B-MAS [13] measures emotion regulation on the basis of Jurist’s [10, 11] theory of mentalized affectivity using three scales: identifying, processing, and expressing. Within the B-MAS, the identifying scale relates to the mentalization of emotions without further differentiation into individual components, whereas the processing and expressing scales relate to emotion regulation. Individual items of the B-MAS can be classified with identifying ("I try to put effort into identifying my emotions."), processing ("I rarely think about the reasons behind why I am feeling a certain way.") as well as communicating emotions ("If I feel something, I will convey it to others.").

The current self-report measures of mentalization lack specific components that are central to the construct such as the assessment of mentalizing emotions with the exception of the B-MAS. However, the B-MAS focuses strongly on emotion regulation and neglects the assessment and differentiation of identification, processing, and communication of mentalizing emotions. Furthermore, it does not differentiate between mentalizing oneself’s and others’ emotions and thus misses a core idea of mentalizing such as the interpersonal component.

The aim of this study is to develop and validate a new self-report questionnaire for the assessment of mentalizing emotions: The newly developed Mentalizing Emotions Questionnaire (MEQ) focuses on the process of mentalizing emotions in terms of identifying, processing, and communicating emotions, distinguishing between self and other. This offers the chance to assess and track changes in the mentalization ability as a process prior to, during and/or after, yet distinct from, emotion regulation.

## Study 1

### Methods and materials

#### Participants

The study was approved by the ethics committee of the Faculty of Behavioral and Cultural Studies at Heidelberg University (AZ Tau 2020 1/1). The online sample was recruited in February 2022 via the panel provider Respondi and conducted via SoSciSurvey [37]. Participants were informed about the study purpose and procedure and provided online written informed consent. After intensive data cleaning, the final *N* comprised 510 participants (50.0% female, 49.4% male, 0.6% diverse), with an age ranging from 18 to 65 years (mean age = 43.3; *SD* = 13.8). Most of the participants did not suffer from mental disorder during the last year (75.1%). Regarding the work situation 68.0% of the participants were employees, 6.9% were self-employed, 12.7% were job-seeking, 9.4% were students and 1.6% were in training. Furthermore, with reference to the highest educational attainment 4.7% had a middle school diploma, 30.8% had a high school diploma or similar, 28.8% completed apprenticeship, 34.3% had a university degree, 1.0% had a PhD and 0.4% were currently going to school.

#### Questionnaire development

The MEQ was developed with reference to the gold standard of mentalizing assessment, the RF Scale, with its original definition of mentalization markers in interview transcripts [16].

Mentalizing emotions was defined in the questionnaire design as follows:

1. Identifying emotions involves perceiving, recognizing, and naming emotions.
2. Processing emotions describes deeper processing and understanding including causes and mental/contextual reasons behind emotions.
3. Communicating emotions means expressing externally as sharing emotions with others.

In each of these components of mentalizing, aspects of interest/curiosity and acceptance as well as multi-perspectives and development-perspectives are of high importance as they are facets of mentalizing according to the RF Scale [1, 16]. Furthermore, the process of mentalizing emotions is operationalized as a self-reflective ability as well as an interpersonal ability (self and other component).

The Mentalizing Emotions Questionnaire (MEQ; S1 File) was developed in a multi-stage, peer reviewed consensus approach. In a first step, items were formulated for the dimension self and other considering the three components of mentalizing emotions: identifying, processing, and communicating. Regarding the dimensions of self and other for each of the three components, items were formulated, respectively for the four following aspects of mentalizing that describe typical processes of mentalizing: interest, acceptance, multi-perspective. and development-perspective [1] (S1 Table). The items include various phrases related to the mentalizing aspects, such as typical behavior, preferences, attitudes, and self-estimated abilities. Per dimension, component and aspect dual items (two items with different phrasing) were used. This step was conducted by three MBT experts (LK, SH and ST are certified MBT therapists and certified RF-raters, ST is also a certified MBT trainer and supervisor). In a second step, plausibility and phrasing of the items were examined by three clinical experts and recommended changes were included. In a further step, to test for social desirability, a group of participants (*N* = 30) rated the dual items in terms of the valence of a person’s characteristic on a scale ranging from very negative (1), either / or (3) to very positive (5). It can be assumed that mentalizing emotions itself is a socially desirable skill, however in order to allow for an as unbiased as possible scoring of the items, the item phrasing regarding social desirability was investigated. Social desirability scores were compared between dual items and the one indicating greater positive or negative valence was removed. The wording of the items was adapted by simplifying and structuring them in a similar way (sentence length, auxiliary words, etc.) and one item was deleted for content reasons. Finally, a pool of 23 items was identified to be tested in the study (S2 Table). In the course of the development of the questionnaire the response format was revised, whereas a 7-point frequency scale was used ranging from never (1), almost never (2), sometimes (3), half of the time (4), often (5), and almost always (6) to always (7). For interpretation of the MEQ a sum score is formed.

#### Data analysis

Data analyses were performed using R Studio [38]. *N* = 177 participants did not finish the questionnaire and were consequently excluded from further analyses. To ensure data quality [39], two instructed response items were included into the dataset (e.g. “If you are attentive, please answer ´very much´.”). *N* = 483 participants answered one of the instructed items incorrectly and were thereby excluded from further analyses. Exceeding data cleaning consisted of the examination of three response anomalies. First, careless responders, defined by either an excessively fast response time (measured in absolute and relative terms) or contradictory responses to the items by always selecting the same answer category were excluded from the data set (*N* = 79). Second, participants with missing data within the central measure (MEQ) were removed (*N* = 1). Lastly, a multivariate outlier analysis was performed resulting in the exclusion of *N* = 28 participants and thereby in a final dataset of *N* = 510 (S2 File). A comparison of the included and excluded participants showed no difference between the groups regarding age (*t* (1255) = -.30, *p* = .77). However, concerning the gender difference between the groups, it was shown that the sample of excluded participants consisted of more men than expected (Fisher’s Exact Test: *p* < .001). In addition to descriptive and preparatory analyses, an Exploratory Factor Analysis (EFA) (using the lavaan package [40]) was performed to examine the given factorial structure of the MEQ. Concerning the evaluation of difficulty (good values between 0.2 and 0.8) and discrimination (acceptable from 0.3), the cuttoffs of Bortz and Döring [41] were used. Within the EFA the decision rules were based on Osborne, Costello [42] for communalities (good values < .4) and Tabachnick and Fidell [43] regarding possible crossloadings. The resulting factor structure was also examined using a descriptive analysis.

### Results

#### Descriptive and preparatory analyses

The descriptive values of the MEQ items are depicted in Table 1. The results of the descriptive analyses show good values for item difficulty (between 0.2 and 0.8) and discrimination (acceptable from 0.3) in almost all cases [41]. Only item 3 (“I accept my emotions as they are.”) did not show satisfactory values (discrimination = .17) and was excluded from further analyses as follows. The normal distribution assumption of the MEQ items could not be confirmed, which is why a robust estimator (minimum residual solution (minres)) is used for the following factor analysis.

**Table 1:** Descriptive values of the MEQ items.

Item No.	Mean	SD	Difficulty	Discrimination	Skewness	Kurtosis
1	5.34	1.34	.62	.64	-.58	2.66
2	4.24	1.48	.46	.43	-.08	2.29
3	4.82	1.29	.55	.17	-.35	2.48
4	3.74	1.37	.39	.55	.13	2.44
5	5.24	1.46	.61	.74	-.67	2.71
6	4.96	1.49	.57	.76	-.61	2.70
7	5.25	1.47	.61	.76	-.79	3.02
8	4.50	1.34	.50	.67	-.27	2.65
9	3.57	1.75	.37	.72	.32	2.13
10	3.84	1.61	.41	.65	.07	2.21
11	4.19	1.70	.46	.72	-.08	2.08
12	3.85	1.63	.41	.72	.06	2.08
13	5.08	1.32	.58	.71	-.56	2.92
14	4.43	1.32	.49	.67	-.23	2.63
15	4.72	1.47	.53	.77	-.43	2.68
16	4.22	1.27	.46	.58	.03	2.66
17	4.75	1.36	.54	.68	-.53	2.84
18	4.48	1.49	.50	.80	-.35	2.61
19	4.57	1.34	.51	.77	-.27	2.40
20	4.37	1.55	.48	.80	-.16	2.52
21	3.56	1.52	.37	.72	-.18	2.29
22	4.21	1.63	.46	.80	.29	2.37
23	3.73	1.64	.39	.74	-.17	2.15

*Mean* = arithmetic mean. *SD* = standard deviation.

*Exploratory factor analysis*. Initially, the number of factors was examined by the empirical Kaiser Criterion, which suggested three factors, as well as a scree plot, which suggested one or three factors. From a theoretical perspective, subdimensions can be considered valuable, which is why we decided to use three factors. Through the subsequent examination of a 3-factor structure with an oblimin rotation, a total of four further items had to be excluded. Items 2, 4, and 16 had to be excluded due to their insufficient communalities (< .4) [42] (S2 Table). Item 22 on the other hand showed too high cross loadings [43] and also had to be removed from further analyses. Thereupon, another factor analysis with oblimin rotation was performed, in which items 21 and 23 showed too high side loadings [43] and were accordingly excluded from further analyses. The final model contains 16 items and three correlating factors with an eigenvalue of > 1 (factor 1: 3.13; factor 2: 3.01; factor 3: 4.34) which explain 65% of the variance. The individual, standardized factor loadings can be obtained from Table 2. Correlations between the three factors show strong values of .63 ≤ *r* ≤ .74.

**Table 2:** Final 16 items of the MEQ and their individual factor loadings.

		Factor		
Item No.	Item	1	2	3
		**Self**		
01	I am interested in my feelings.	.54		
05	I am interested in understanding my feelings.	.79		
06	I try to understand the different reasons for my feelings.	.90		
07	I think it is helpful to understand the causes of my feelings.	.93		
08	With some distance, I can understand my feelings in a new way.	.48		
			**Communicating**	
09	I think it is exciting to talk with others about my feelings.		.75	
10	I can explain my different feelings to others.		.79	
11	I think it is useful to talk about my feelings.		.77	
12	I can talk to others about how my feelings change.		.96	
				**Other**
13	I am interested in the feelings of others.			.69
14	I can perceive conflicting feelings in others.			.69
15	I think it is enriching to recognize feelings in others.			.71
17	I try to see situations through the other person’s eyes.			.83
18	I find it helpful to think about the reasons for others’ feelings.			.91
19	Through time, I can better understand the feelings of others.			.81
20	I think it is exciting to think about where others’ feelings come from.			.71

Skewness of the MEQ overall scale and its subscales was between -.07 and .01, the kurtosis of the MEQ overall scale and its subscales was between 2.20 and 3.04. The mean value and standard deviations were as follows: overall scale: 73.14 (*SD* = 17.58), factor 1: 25.29 (*SD* = 5.97), factor 2: 15.45 (*SD* = 5.92), factor 3: 32.40 (*SD* = 8.09). There were no indications of floor- or ceiling effects.

The overall scale (mentalizing emotions) consists of 16 items within three factors (self, communicating, and other):

Factor 1 (self) consists of five items: The factor describes mentalizing emotions in the dimension self with the components identifying and processing. Identifying and processing emotions considers hereby the aspects of interest, acceptance, multi-perspective, and development-perspective.

Factor 2 (communicating) contains four items: The factor describes mentalizing emotions in the dimension self with the component communicating. Communicating emotions towards others considers hereby the aspects of interest, acceptance, multi-perspective, and development-perspective.

Factor 3 (other) consists of seven items: The factor describes mentalizing emotions in the pole others with the components identifying and processing. Identifying and processing emotions of others consider hereby the aspects of interest, acceptance, multi-perspectives, and development-perspective.

## Study 2

### Methods and materials

#### Participants

The study was approved by the ethics committee of the Faculty of Behavioral and Cultural Studies at Heidelberg University (AZ Tau 2020 1/1-A1). Analogously to the first study, the sample of the second study was recruited via the panel provider Respondi (April 2023) and conducted via SoSciSurvey [37]. Participants were informed about the study purpose and procedure and provided online written informed consent. The sample size after data cleaning was *N* = 509 (53.3% female, 46.2% male, 0.6% diverse), with age ranging from 18 to 65 years (mean age = 44.0; *SD* = 13.2). Most of the participants did not suffer from mental disorder during the last year (74.1%). 78 of the 132 participants (59.1%) with a mental disorder within the last year went into treatment, whereby 43.2% were in outpatient treatment.

69.1% of the participants were employees, 7.7% were self-employed, 12.8% were job-seeking, 8.5% were students, and 2.0% were in training. Furthermore, with reference to the highest educational attainment 16.3% had a middle school diploma, 15.0% had a high school diploma or similar, 28.7% completed apprenticeship, 38.6% had a university degree, 1.2% had a PhD, and 0.4% were currently going to school.

No significant difference was found considering age, gender, and educational status between study 1 and 2 samples using a two-sample t-test.

#### Measures

In Study 2, the MEQ was presented in its 16-item final form obtained from study 1. To validate the MEQ the following questionnaires were employed:

#### Reflecting Functioning Questionnaire (RFQ)

The RFQ-8 [44] is an 8-item self-report measure of mentalizing. In order of recent recommendations [19, 45], the mean score of a psychometrically optimized six-item version of the scale (RFQ-6) [19] was used. The shortened version captures the level of uncertainty about mental states (i.e., hypomentalizing). The items are rated on a 7-point scale ranging from *strongly disagree* (1) to *strongly agree* (7). The questionnaire’s Cronbach’s alpha in the current study was .81.

#### Certainty about mental states Questionnaire (CAMSQ)

The CAMSQ [21] is a 20-item self-report measure of mentalizing capturing the certainty about mental states. The questionnaire consists of two scales: Self and Other. The items are rated on a 7-point scale ranging from *never* (1) to *always* (7). The subscales’ Cronbach’s alpha in the current study were .92-.93.

##### Mentalizing questionnaire (MZQ)

The MZQ [20] is a 15-item self-report measure for mentalizing. It is operationalized by four aspects associated with mentalizing: emotional awareness, regulation of affect, psychic equivalence mode, and refusing self-reflection. The items are rated on a 5-point scale ranging from *no agreement at all* (1) to *total agreement* (5), whereas high scores indicate less mentalizing. The subscales’ Cronbach’s alpha in the current study were .65-.76 and of the overall scale .86.

#### Attributional Complexity Scale (ACS)

The ACS [31] is a self-report measure that assess attributional complexity. In this study a short form of the ACS [46] with seven items was chosen. The items are rated on a 7-point scale ranging from *not true at all* (1) to *accurately true* (7). The questionnaire’s Cronbach’s alpha in the current study was .88.

#### Epistemic Trust, Mistrust and Credulity Questionnaire (ETMCQ)

The ETMCQ [30] is a self-report measure for three scales: Epistemic Trust, Mistrust, and Credulity. In this study the German Version of the ETMCQ [47] with 15 items was used. The items are rated on a 7-point scale ranging from *strongly disagree* (1) to *strongly agree* (7). The subscales’ Cronbach’s alpha in the current study were .69-.78.

#### Brief-Mentalized Affectivity Scale (B-MAS)

The Brief-MAS [13] is a short form of the original 60-item self-report measure [48] to assess emotion regulation based on the Theory of Mentalized Affectivity [10, 11]. The three-component structure of the MAS could not be replicated [49, 50], whereas the three-component structure of the B-MAS could be replicated [50, 51]. The B-MAS consists of three subscales (identifying, processing, expressing), whereas the 12 items are rated on a 7-point scale ranging from *strong rejection* (1) to *strong agreement* (7*)*. The subscales’ Cronbach’s alpha in the current study were .20-.30. These scores are unacceptably low, which is why the B-MAS was only reported with reservation in the correlation analyses.

#### Empathy Quotient (EQ)

The EQ [35] is a 40-item self-report measure of empathy with three subscales: cognitive empathy, emotion reactivity, and social skills. Items are rated on a 4-point scale ranging from *strongly disagree* (1) to *strongly agree* (4). The subscales’ Cronbach’s alpha in the current study were .67-.88 and of the overall scale .87.

#### Emotion Regulation Questionnaire (ERQ)

The ERQ [52] is a 10-item self-report measure for emotion regulation. It assesses emotion regulation with the two scales reappraisal and suppression. The items are rated on a 7-point scale ranging from *not true at all* (1) to *perfectly true* (7). In this study the German Version of the ERQ [53] was used. The subscales’ Cronbach’s alpha in the current study were .78-.88.

#### Emotion Belief Questionnaire (EBQ)

The EBQ [32] is a 16-item self-report measure for beliefs about emotions consisting of three subscales: general controllability, usefulness of positive, and negative emotions. The items are rated on a 7-point scale ranging from *does not apply at all* (1) to *completely true* (7). In this study the German Version of the EBQ [54] was used. The subscales’ Cronbach’s alpha in the current study was .80-.88 and of the overall scale .89.

#### Geneva Emotion Knowledge Test–Blends Brief Form (GEMOK-Blends)

The brief form of the GEMOK-Blends [33] is a 10-item task-based measure of emotion recognition. The tasks are based on text descriptions of scenarios involving two emotional experiences of a target person. Per task there are five pairs of terms as response options, whereas the best description of the targets mental states needs to be chosen. The task’s Cronbach’s alpha in the current study was .48. This score is unacceptably low, therefore the GEMOK-Blends was removed from the correlation analyses due to poor psychometric performance.

#### Level of Personality Functioning Scale–Brief Form 2.0 (LPFS-BF)

The LPFS-BF [55, 56] is a 12-item self-report measure of personality functioning. Impairments in personality functioning are measured with the two scales self-functioning and interpersonal functioning. The items are rated on a 4-point scale ranging from *completely untrue* (1) to *completely true* (4), whereas high scores indicate dysfunction. The subscales’ Cronbach’s alpha in the current study was .77-.88 and of the overall scale .89.

#### Symptom-Checklist K9 (SCL-K-9)

The SCL-K9 [27] is a nine-item self-report measure of symptom distress experienced in the past week. It is a short form of the SCL [57, 58]. Items are answered on a 5-point scale ranging from *not at all* (0) to *extremely* (4). The questionnaire’s Cronbach’s alpha in the current study was .90.

#### Short Alexithymia Scale (SAS-3)

The SAS-3 [26] is a three-item self-report measure for alexithymia. It is a short form of the Toronto Alexithymia Scale [59]. The items are rated on a 5-point scale ranging from *not true at all* (1) to *always true* (5). The questionnaire’s Cronbach’s alpha in the current study was .66.

#### Berlin Test for the Assessment of Fluid and Crystallized Intelligence—Short Form Crystallized Intelligence (BEFKI GC-K)

The BEFKI GC-K [29] is a task-based measure for assessing declarative knowledge with 12 questions and four response options per question. The task is composed of questions from various areas as natural sciences, humanities, and social sciences. The items are in accordance with the definition of crystalline intelligence by Cattell and Carol [60]. The task’s Cronbach’s alpha in the current study was .60.

*Data analysis*. Data analyses were performed using R Studio [38]. Three instructed response items were included in the second study to ensure data quality [39] (e.g. “If you are attentive, please answer ´very much´.”). *n* = 216 participants answered the instructed item incorrectly and were thereby excluded from the questionnaire survey. Moreover, a falling below a time limit (900 seconds) was already specified as an exclusion criterion in the questionnaire survey. Data cleaning of *N* = 573 participants, who have successfully completed the questionnaire survey, consisted of identifying and excluding careless responders who were characterized by an unrealistically fast response time [61]. For this purpose, the relative speed index (RSI), a variable conducted by SoSciSurvey [37], was used (excluding participants with an RSI ≥ 2) [62] (*n* = 42) as well as the total processing time (excluding participants with less than half the average processing time) (*n* = 16). Furthermore, a cut-off time for the minimum speed of the MEQ response [63] was used as an exclusion criterion (excluding participants answering the MEQ within less than 30 seconds) (*n* = 6). After data cleansing, the survey sample consisted of *N* = 509 participants (S3 File). The examination of group differences between included and excluded participants revealed no significant results with regards to age (*t* (911) = -1.14, *p* = .25) and gender (Fisher’s Exact Test: *p* = .26).

Confirmatory Factor Analysis was performed by the R package lavaan [40]. To assess the model fit Comparative Fit Index (CFI), Root Mean Square Error of Approximation (RMSEA), and Standardized Root Mean Square Residual (SRMR) were used. The cut-offs for these indices were dynamically calculated [64].

To assess scale reliability, we used Cronbach’s alpha: 0.6 < *α* < 0.7 indicates an acceptable level of reliability, an *α* ≥ 0.8 indicates an excellent level, whereas values higher than 0.95 indicates a possible redundancy [65].

To assess the validity and correlates, we firstly used Pearson correlation coefficient (*r*): according to Cohen [66] *r* = .10 is considered as small, *r* = .30 as medium and *r* = .50 as large in magnitude. Validity was assessed using the constructs of mentalization, empathy, epistemic trust, emotion regulation, emotion recognition, perceived controllability, and usefulness of emotions and mentalized affectivity, which are closely related to mentalizing emotions. Mentalization was assessed with the RFQ-6, CAMSQ, MZQ, and ACS. Epistemic trust was assessed with the ETMCQ. Focusing on emotions, mentalized affectivity was assessed with the B-MAS, empathy with the EQ, emotion regulation with the ERQ, beliefs about emotions with the EBQ, and emotion recognition with the GEMOK-Blends. As psychopathological correlates personality functioning (LPFS-BF), general psychological distress (SCL-K-9), and symptoms of alexithymia (SAS-3) were used. To test the incremental validity of the MEQ, a Structural Equation Model was performed. Thereby, CAMSQ, MZQ, RFQ-6, and MEQ were used as exogenous and ERQ and EQ as endogenous variables. To evaluate the model fit we used the following cut-offs for an acceptable fit: Comparative Fit Index (CFI) ≥ .9; Root Mean Square Error of Approximation (RMSEA) ≤.08; Standardized Root Mean Square Residuals (SRMR) ≤.08 [67]. Furthermore, crystalline intelligence (BEFKI GC-K) was assessed. For the calculation of the correlation with the BEFKI GC-K, a subsample (*n* = 420) was formed by setting the time of completion of the BEFKI GC-K to a maximum of 5 minutes (300 seconds).

To analyze whether MEQ values differ between a healthy sample (persons with non-pre-existing mental disorders in the last year) and people with mental disorders in the last year an unpaired Welch’s t-test was calculated. Furthermore, to investigate whether gender (female and male) plays a role in relation to the MEQ and whether there are gender differences in the values of the MEQ an unpaired Welch’s t-test was calculated. Due to the small number of participants with the indication diverse (*n* = 3), diverse could not be considered in the analysis. To test the one-sided effect of age on the MEQ a linear regression was calculated.

### Results

#### Confirmatory Factor Analysis

**Fig 1:**
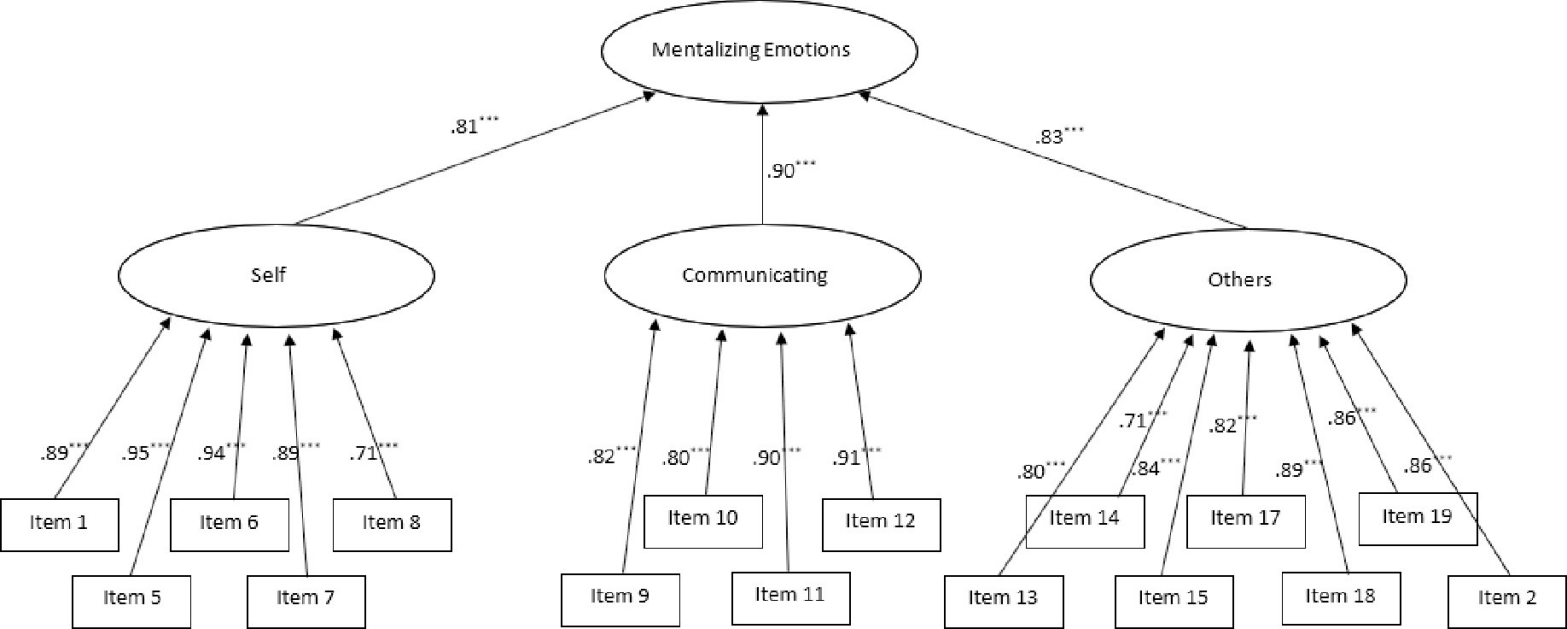


The 3-factorial structure postulated by the exploratory factor analysis in study 1 was supported in the Confirmatory Factor Analysis. The model fit indices showed the following results: CFI = .959, RMSEA = .078, SRMR = .04. Calculation of the dynamic fit indices showed that the cut-offs for a good model fit were SRMR ≤ .04, RSMEA ≤.074, and CFI ≥ .966, and the values for an acceptable fit were SRMR ≤.046, RSMEA ≤.102, and CFI ≥ 944. Accordingly, the SRMR corresponds to a good model fit and the RMSEA and CFI to an acceptable model fit. Consequently, the model can be accepted. The final model of the MEQ confirmed by the CFA, including the standardized factor and item loadings, is shown in Fig 1.

#### Empirical distributions, reliability, and association with age, gender, and mental disorder

Skewness of the MEQ overall scale and its subscales was between -.78 and .06 and the kurtosis of the MEQ overall scale and its subscales was between 2.31 and 3.12. The mean value and standard deviations were as follows: overall scale: 74.8 (*SD* = 19.15), factor 1: 25.89 (*SD* = 6.75), factor 2: 15.98 (*SD* = 6.11), factor 3: 32.94 (*SD* = 9.04). There were no indications of floor- or ceiling effects.

Internal consistency measured with Cronbach’s alpha reliability coefficient showed excellent values for factor 1 (.94), factor 2 (.92) and factor 3 (.94) as well as for the overall scale (.95).

Unpaired Welch’s t-test indicated a difference between gender on the MEQ, *t* (468.6) = 3.75, *p* < .001. Female participants (*M* = 77.83, *SD* = 17.71) showed a significant higher mean of the MEQ total score than male participants (*M* = 71.44, *SD* = 20.26).

A simple linear regression with MEQ as the dependent variable and age as the explanatory variable was significant, *β* = -.40, *t* (504) = -6.51, p < .001, *F* (1, 506) = 42.43, *p* < .001. 7.74% of the variance from MEQ can be explained by the variable age. With a younger age, the MEQ total score seems to be higher.

Unpaired Welch’s t-test indicated no difference on MEQ (*t* (242.72) = 0.97, *p* = .33) between participants with pre-existing mental disorder in the last year (*M* = 76.15, *SD* = 18.24) versus participants with non-pre-existing mental disorder in the last year (*M* = 74.33, *SD* = 19.46).

#### Validity

MEQ subscales and overall scores showed significant correlations with most of the measures used to assess construct validity (Tables 3 and 4). Most of the significant correlations between MEQ overall scale mentalizing emotions and the other constructs were positive, suggesting associations with the analyzed constructs (.18 ≤ *r* ≤ .69). In the following, only correlations with correlation coefficients greater than .30 or less than -.30 are specified, as these show a moderate effect and therefore are assumed to be values of consideration according to Cohen’s [66] cut-offs. As expected, significant negative correlations were shown between MEQ overall scale plus its subscales and MZQ and its subscales using self-reflection and emotional awareness, ETMCQ mistrust, ERQ suppression, EBQ and its subscales (-.37 ≤ *r* ≤ -.15). No significant correlations were found between MEQ overall scale plus its subscales and RFQ-6, MZQ regulation of affect, and ETMCQ credulity.

**Table 3:** Means, standard deviations, and correlations of the MEQ and mentalization as well as epistemic trust questionnaires.

	Variable	*M*	*SD*	1	2	3	4	5	6	7	8	9	10	11	12	13	14	15
(1)	MEQ overall scale	74.80	19.15															
(2)	MEQ self	32.94	9.04	.88**														
(3)	MEQ communicating	15.98	6.11	.85**	.60**													
(4)	MEQ other	25.89	6.75	.89**	.63**	.60**												
(5)	RFQ	21.97	7.10	-.04	-.03	-.05	-.02											
(6)	CAMSQ self	50.54	10.32	.49**	.48**	.42**	.40**	-.38**										
(7)	CAMSQ other	45.14	9.71	.59**	.45**	.45**	.61**	-.14**	.58**									
(8)	MZQ total score	40.60	9.55	-.16**	-.15*	-.21**	-.09*	.64**	-.10*	-.35**								
(9)	MZQ refusing self-reflection	10.29	3.19	-.35**	-.33**	-.38**	-.24**	.38**	-.11*	-.22**	.77**							
(10)	MZQ emotional awareness	10.35	3.20	-.19**	-.17**	-.22**	-.12**	.57**	-.15**	-.42**	.82**	.53**						
(11)	MZQ psychic equivalence	11.97	3.25	.08	.10*	.01	.10*	.52**	.01	-.21**	.79**	.40**	.51**					
(12)	MZQ regulation of affect	7.98	2.40	-.05	-.04	-.07	-.02	.59**	-.08	-.25**	.79**	.49**	.54**	.55**				
(13)	ACS	33.33	8.57	.69**	.56**	.51**	.69**	.10*	.51**	.32**	.04	-.15**	.01	.21**	.09*			
(14)	ETMCQ trust	24.56	5.14	.56**	.49**	.54**	.46**	.00	.35**	.32**	-.12**	-.28**	-.09	.08	-.10*	.44**		
(15)	ETMCQ mistrust	19.76	5.10	-.11*	-.09	-.15**	-.07	.42**	-.04	-.12**	.61**	.51**	.45**	.45**	.53**	.04	-.25**	
(16)	ETMCQ credulity	15.50	5.40	.04	.08	.05	-.02	.50**	-.07	-.12**	.51**	.33**	.43**	.43**	.42**	.12**	.10*	.48**

*M* and *SD* are used to represent mean and standard deviation, respectively.

* indicates *p* < .05.

** indicates *p* < .01.

**Table 4:** Means, standard deviations, and correlations of the MEQ and emotion related questionnaires.

	Variable	*M*	*SD*	1	2	3	4	5	6	7	8	9	10	11	12	13
1	MEQ overall scale	74.80	19.15													
2	MEQ self	32.94	9.04	.88**												
3	MEQ communicating	15.98	6.11	.85**	.70**											
4	MEQ other	25.89	6.75	.89**	.63**	.60**										
5	EQ total score	38.33	11.12	.44**	.31**	.28**	.50**									
6	EQ cognitive empathy	9.86	4.48	.48**	.33**	.37**	.52**	.72**								
7	EQ social skills	5.65	2.62	.18**	.14**	.15**	.19**	.69**	.40**							
8	EQ emotion reactivity	11.02	4.12	.46**	.36**	.31**	.51**	.86**	.49**	.43**						
9	ERQ reappraisal	26.67	6.91	.32**	.31**	.25**	.26**	.22**	.30**	.19**	.18**					
10	ERQ suppression	15.74	4.94	-.37**	-.35**	-.46**	-.22**	-.27**	-.18**	-.29**	-.27**	.04				
11	EBQ total score	42.45	14.99	-.19**	-.15**	-.12**	-.22**	-.40**	-.15**	-.38**	-.32**	-.07	.34**			
12	EBQ controllability	22.59	9.14	-.15**	-.11**	-.10*	-.17*	-.38**	-.15**	-.38**	-.30**	-.12**	.31**	.92**		
13	EBQ negative	13.26	5.51	-.15**	-.09*	-.09*	-.18*	-.18**	-.09	-.15**	-.12**	.06	.20**	.74**	.50**	
14	EBQ positive	6.60	3.66	-.20**	-.20**	-.12**	-.20**	-.40**	-.14**	-.37**	-.35**	-.06	.34**	.68**	.54**	.28**

*M* and *SD* are used to represent mean and standard deviation, respectively.

* indicates *p* < .05.

** indicates *p* < .01.

In Table 3 correlations between MEQ and mentalizing constructs (RFQ-6, CAMSQ, MZQ, ACS) as well as epistemic trust (ETMCQ) are shown. MEQ overall scale and its three subscales were associated the strongest with CAMSQ self and other, ACS, and ETMCQ subscale trust (.40 ≤ *r* ≤ .69), whereas correlations with MZQ subscale refusing self-reflection was less distinct. Noteworthy is that MEQ subscale self correlated strongly with mentalizing the self measured by the CAMSQ self (*r* = .48), whereas MEQ subscale other correlated strongly with mentalizing other, measured by the CAMSQ other (*r* = .61).

In Table 4 correlations between MEQ and emotion constructs (EQ, ERQ, EBQ) are shown, demonstrating the expected correlation directions. MEQ overall scale showed the strongest correlates to empathy measured by EQ total score, EQ cognitive empathy, and EQ emotion reactivity (.44 ≤ *r* ≤ .49), whereas correlations to emotion regulation were less distinct. MEQ subscale self was the strongest associated to empathy (EQ total score, EQ cognitive empathy, EQ emotion reactivity; .31 ≤ *r* ≤ .36) and emotion regulation (ERQ reappraisal, *r* = .31; ERQ suppression, *r* = -.35). MEQ subscale communicating correlated the strongest with ERQ suppression (*r* = -.46) and correlated less pronounced with EQ subscale cognitive empathy and EQ subscale emotion reactivity. MEQ subscale other correlated the strongest with empathy (EQ total score, EQ cognitive empathy, EQ emotion reactivity;.50 ≤ *r* ≤ .52). MEQ overall scale and its subscales showed a small to medium correlation to EBQ total score and its subscales.

Due to the inacceptable low internal consistency and therefore poor psychometric performance of the B-MAS, the correlations between the B-MAS and the MEQ should only be interpreted with caution. The MEQ overall score shows medium to large correlations with the B-MAS subscales identifying (*r* = .49), processing (*r* = .36), and expressing (*r* = .34). The MEQ subscale self correlated moderately to largely with the B-MAS subscale identifying (*r* = .47), processing *r* = .33), and expressing (*r* = .30). Between the MEQ subscale communicating and the B-MAS subscales the following small to medium and medium to large correlation were shown: identifying (*r* = .40), processing (*r* = .28), and expressing a (*r* = .25). The MEQ subscale other showed medium to large associations to the B-MAS subscales identifying (*r* = .41), processing (*r* = .31), and expressing (*r* = .32). When analyzing the correlation of the MEQ and the B-MAS, it is noticeable that the correlations with the B-MAS subscale identifying are consistently the strongest.

To test incremental validity, a structural equation model was created in which a regression of the previous mentalizing questionnaires (CAMSQ, MZQ, RFQ-6) and the MEQ on emotion regulation (ERQ) and empathy (EQ) was presented. This method was chosen to reduce the type 1 errors that often arise [68]. However, the structural equation model conducted in this study did not achieve an acceptable fit. This might be caused by the relatively low degrees of freedom. Nevertheless, the model was not possible to interpret. Consequently, it was decided to use multiple regression to calculate incremental validity. To limit the risk of type 1 error, it was decided to consider only one dependent variable. Thereby, empathy was included as the dependent variable. To calculate incremental validity, the multiple regression was run once with and once without the predictor MEQ. The increase in the explained variance (R^2^) could thus be attributed to the addition of the MEQ. The increase from the model without MEQ (R^2^ = .30, *F*(3, 505) = 72.99, *p* < .001)) to the model with MEQ (R^2^ = .35, *F*(4, 504) = 67.88, *p* < .001) was R^2^ = .05, which equals 5% of the total variance. As the significance of the increase in variance is not calculated automatically in our procedure, it was determined in a further regression analysis. For this purpose, the residuals of the model without the MEQ were used as the dependent variable and the MEQ score as the predictor. The result shows a significant model (*R*^2^ = .04, *F*(1, 507) = 21.46, *p* < .001) and association (*b* = .10, *p* < .001) Thus, incremental validity of the MEQ can be assumed.

#### Associations to psychopathology and crystalline intelligence

As shown in Table 5, there were only isolated significant correlations between the MEQ and its subscales and psychopathology and crystalline intelligence. MEQ overall scale and its subscales correlated strongest with alexithymia (SAS-3, -.52 ≤ *r* ≤ -.34), but also with personality functioning (LPFS-BF total score, LPFS-BF interpersonal), symptom distress (SCL-K-9), and crystalline intelligence (BEFKI GC-K). For the correlation calculations of the BEFKI GC-K and MEQ a subsample was formed (*n* = 420), since the time criterion (< 300 seconds) of the BEFKI GC-K was considered.

**Table 5:** Means, standard deviations, and correlations of the MEQ and psychopathological and intelligence correlates.

	Variable	*M*	*SD*	1	2	3	4	5	6	7	8	9
(1)	MEQ_overall scale	74.80	19.15									
(2)	MEQ self	32.94	9.04	.88**								
(3)	MEQ communicating	15.98	6.11	.85**	.70**							
(4)	MEQ other	25.89	6.75	.89**	.63**	.60**						
(5)	LPFS-BF total score	23.22	7.30	-.10*	-.08	-.12**	-.06					
(6)	LPFS-BF self	11.85	4.50	-.05	-.06	-.09*	.01	.94**				
(7)	LPFS-BF interpersonal	11.37	3.43	-.14**	-.10*	-.13**	-.15**	.90**	.69**			
(8)	SCL-K-9	18.43	7.78	.08	.06	.01	.12**	.68**	.53**	.69**		
(9)	SAS-3	8.19	2.68	-.46**	-.38**	-.52**	-.34**	.51**	.48**	.46**	.38**	
(10)	BEFKI GC-K	8.95	2.11	-.11*	-.11*	-.11*	-.09	-.09	-.06	-.10*	-.22**	.29**

*M* and *SD* are used to represent mean and standard deviation, respectively.

* indicates *p* < .05.

** indicates *p* < .01.

## General discussion

The aim of this research was to develop and evaluate a self-report measure to assess mentalizing of emotions with a new questionnaire: the Mentalizing Emotions Questionnaire (MEQ; S1 File). In both validation studies, the MEQ showed acceptable to good psychometric properties, with a clear and theoretically relevant factor structure, a very high internal consistency and good construct validity. Mentalizing emotions incorporates components as identifying, processing, and communicating of emotions [10, 11]. As a reflection of this, the EFA in study 1 indicated a three-factor structure consisting of 16-items with an overarching factor that was confirmed by the Confirmatory Factor Analysis in study 2. The overall scale mentalizing emotions summarizes the three factors: self, communicating, and others. Mentalizing emotions of the self and others includes perceiving, recognizing, and naming emotions (identifying) as well as a deeper process and understanding including causes and mental or contextual reasons behind emotions (processing). Communicating mentalized emotions of the self refers to sharing and discussing own emotions with others (communicating). All three subscales include mentalization aspects such as interest, acceptance, multi-perspective-taking, and development-perspectives [16].

The MEQ was constructed similarly to the B-MAS with the components identifying, processing, and communicating [10, 11, 13, 48]. The main difference between the MEQ and the B-MAS is that the MEQ approaches the concept of emotion regulation in the sense that understanding emotions leads to emotion regulation, whereas the B-MAS views mentalizing emotions as a part of the emotion regulation process. The MEQ focuses on mentalizing emotions as an effective form of emotion regulation involving self and others, whereas the B-MAS refers to the regulation of emotions including aspects of mentalizing emotions of the self while excluding others. In this sense, the scale identifying of the B-MAS, which refers to mentalizing emotions, is represented in the components identifying and processing in the MEQ. With bearing the poor psychometric performance of the B-MAS within this study in mind, it was shown that the B-MAS subscale identifying correlates most strongly with the MEQ and its subscales in comparison to the other B-MAS subscales processing and expressing. For example, aspects such as “understanding the meaning of emotions in the context of individual circumstances and exploring the source of emotions” [13] as part of identifying in the B-MAS was defined as a component of processing in the MEQ. The theoretical construction of the MEQ was based on Fonagy’s mentalization theory [1, 4] and its operationalization using the RF Scale [16]. While the B-MAS displayed excellent psychometric properties during its initial validation, the deficient psychometric values discovered in this study are noteworthy and bring into question the B-MAS’s utility. Thus, an assessment of the psychometric quality of the B-MAS is necessary.

The construct Mentalizing Emotions in MEQ is newly redefined, so that the previous questionnaires do not directly measure convergent or discriminant validity, but the extent to which parts of the concept are related to other constructs. Associations between the MEQ and pre-existing mentalizing and epistemic trust as well as emotion related questionnaires provided evidence for the MEQ’s validity. The MEQ shows small to large effects associated with the majority of the questionnaires tested and in the expected direction. The MEQ is seen as an important sub-construct of mentalization, whereas with the solely focus on emotions as well as self and others not too large correlations with the mentalization questionnaires were expected. Furthermore, the MEQ overlaps with constructs of emotion-related questionnaires, but again not too large correlations were expected, as MEQ captures more than just one facet. In terms of incremental validity, there was a 5% increase in the variance of empathy due to the inclusion of the MEQ. This increase in explained variance can be interpreted as an indication of incremental validity. Unfortunately, due to the unacceptable fit of the structural equation model, only incremental validity with respect to one variable (empathy) could be examined. Thus, a full statement about the incremental validity is not yet possible. This should be implemented in future research. The MEQ total scale mentalizing emotions and its subscales self, communicating and other showed medium to large associations to certainty about mental states (CAMSQ self and other), attributional complexity (ACS) and epistemic trust (ETMCQ trust) as well as to empathy (EQ total scale, EQ cognitive empathy, EQ emotion reactivity). Small to medium negative associations were found between the MEQ scales and the MZQ scale refusing self-reflection, which captures avoiding thinking about mental states [20]. Furthermore, there were small to medium associations between the MEQ scales and emotion regulation, whereas positive associations were shown towards emotion reappraisal and negative associations to emotion suppression. Likewise, a small negative association was found between the MEQ scales and beliefs about emotions (EBQ total scale, EBQ positive emotions), implicating a discrepancy between mentalizing emotions and the belief about usefulness of positive emotions. However, it was noticeable that there was no connection between the MEQ and the RFQ-6 as well as the MZQ subscales regulation of affect and only small to medium associations between the MEQ total score and the MZQ subscale psychic equivalence mode as well as subscale emotional awareness. These results could be explained by the constructs used in the RFQ-6 and MZQ that were designed to detect hyper- or hypomentalization in contrast to the MEQ. Furthermore, the RFQ-6 focusses mainly on cognition [44], whereas the MEQ concentrates on emotions.

Focusing on the MEQ subscales: expected medium to large associations were found between mentalizing emotions of the self and certainty about mental states of the self (CAMSQ self) as well as mentalizing emotions of others and certainty about mental states of others (CASMQ other). Additionally, mentalizing emotions of others showed large associations with empathy (EQ total score, EG cognitive empathy, EQ emotion reactivity) and therefore, giving some evidence for the construct independence of the respective subscales. Furthermore, an expected medium negative association between communicating emotions of the self and emotion suppression (ERQ) was found.

There were none to small negative associations between the MEQ and its subscales with personality functioning (LPFS) and none to small positive associations with symptom severity (SCL-K-9). In addition, there were no differences in the MEQ and its subscales between participants suffering of a mental disorder in the last year and participants with no pre-existing mental disorder in the last year. Both results may be mainly influenced by the convenience sample with very low psychopathology in general. As previously deficits in mentalizing have been linked to mental disorders [69], whereas improvements in patients’ mentalizing have been linked to general improvement in psychological functioning [70–73]. However, the MEQ differentiated between participants with alexithymia (SAS-3), which assesses emotional blindness. Between alexithymia and the MEQ and its subscales, particularly communicating, medium to high associations were found.

There was only a small negative correlation with crystalline intelligence, implying an independence of the two constructs. It should also be noted that only crystalline intelligence is measured, which is related to general knowledge and the corresponding level of education.

There was a gender difference in MEQ between women and men, with women appearing to have a higher subjective ability to mentalize emotions. In addition, the age of the participants had an influence on the MEQ implicating with younger age the ability to mentalize emotions to be higher.

In summary, the MEQ is a questionnaire that is closely operationalized to the mentalization concept of the RF Scale with a focus on identifying, processing and communicating emotional states differentiated into self and others. Further, the MEQ is closely related to epistemic trust and empathy as well as aspects of emotion regulation. Rather low associations were found with psychopathological characteristics. All in all, the MEQ is the first questionnaire to explicitly measure mentalizing emotions, divided into mentalizing one’s own emotions, communicating about emotions of the self and mentalizing about emotions of others. In the first study, items related to talking to others about their emotions were also included, but these items were removed from the model due to insufficient loading.

### Limitations and perspective

Recruiting the samples via a panel provider is economical, but bears the risk of limited generalizability to the study results. It should be considered that the factor loadings between the first and second sample differ unexpectedly in some cases (e.g. item 1: b = .54 in sample 1; b = .89 in sample 2). However, the calculations regarding sample differences did not yield any significant results, so that a conclusion of the different factor loadings on the sample compositions is not possible. Nevertheless, the factor structure should be checked again in an external sample to make sure that the samples really do not have any effect on the factor structure. Furthermore, it can be discussed, if the internal consistency of the overall scale (α = .95) is a reference for redundancy based on Eisinga, Grotenhuis [65] implicating scores above .95 as potential redundant. This should be verified and if necessary a brief form of the MEQ can be created. Regarding incremental validity, the results should be interpreted against the background of a possible type 1 error bias. Further, it should be considered that the structural equation model did not reach an acceptable fit. In future studies, the incremental validity should be tested again, maybe regarding other desirable measures, especially a validation with the RF Scale [16]. Moreover, the convergent validity using the RF Scale should be tested: the MEQ itself is a self-report measure and therefore captures a subjective mentalizing ability, whereas self-report of mentalizing are more consistent with measuring a specific mentalizing self-concept rather than an actual ability [21]. A limitation is that the MEQ was validated with self-reports only. Additionally, self-report methods in general are unlikely to capture actual differences in ability as shown as a lack of convergent validity between self-reports and other methods [74–78]. Therefore, we recommend that all mentalizing questionnaires showing good parametric values should be tested on the RF Scale to determine their validity. Furthermore, the retest reliability of the MEQ was not tested in this research, which should be done in future studies. So far, there is only a small association of the MEQ with psychopathology, but to confirm this, the MEQ should be tested in a patient sample. It would also be interesting to use the MEQ as an assessment tool over the course of psychotherapy to examine psychotherapy processes. A significant limitation of the use of the MEQ is that it is currently only available in German. A translation into English and other languages as well as a validation of this would be desirable and conceivable for the future.

## Conclusion

The MEQ is a valid and reliable questionnaire for the assessment of mentalizing emotions, divided into self, communicating, and other. The MEQ can provide a deeper understanding of how individuals mentalize their own emotions, communicate them, and how they perceive and process the emotions of others. Especially for studies of psychotherapy processes this could provide important insights into how mentalizing emotions evolve.

## Supporting information

S1 File Mentalizing Emotions Questionnaire (MEQ)—German version. (DOCX)

S2 File Data set of Study 1. (XLSX)

S3 File Data set of Study 2. (XLSX)

S1 Table 16-item MEQ divided into poles, elements and aspects. (DOCX)

S2 Table List of the original 23 items. bold = items of the final MEQ.
(DOCX)
