# Supplementary material for: Development and validation of the Mentalizing Emotions Questionnaire: A self-report measure for mentalizing emotions of the self and other
Source: PLoS One. 2024 May 6;19(5):e0300984. doi: 10.1371/journal.pone.0300984 (PMC11073734; doi:10.1371/journal.pone.0300984)
Supplement: S1 File — (DOCX) [file pone.0300984.s001.docx]

Mentalizing Emotions Questionnaire (MEQ) - German Version

Im Folgenden geht es darum, wie sie mit ihren eignen Gefühlen und den von anderen umgehen. Es werden Ihnen verschiedene Aussagen präsentiert, die sich zur Selbstbeschreibung eignen können. Bitte kreuzen Sie für jede Aussage an, welche Antwortoption am besten auf Sie zutrifft, von "nie" über "in der Hälfte der Fälle" zu "immer".

|  | nie | fast nie | manchmal | in der Hälfte der Fälle | oft | fast immer | immer |
| --- | --- | --- | --- | --- | --- | --- | --- |
| 1. Ich interessiere mich für meine Gefühle. | □ | □ | □ | □ | □ | □ | □ |
| 1. Ich bin daran interessiert, meine Gefühle zu verstehen. | □ | □ | □ | □ | □ | □ | □ |
| 1. Ich versuche die verschiedenen Gründe meiner Gefühle zu verstehen. | □ | □ | □ | □ | □ | □ | □ |
| 1. Ich finde es hilfreich, die Ursachen meiner Gefühle zu verstehen. | □ | □ | □ | □ | □ | □ | □ |
| 1. Mit etwas Abstand kann ich meine Gefühle neu verstehen. | □ | □ | □ | □ | □ | □ | □ |
| 1. Ich finde es spannend, mich mit anderen über meine Gefühle auszutauschen. | □ | □ | □ | □ | □ | □ | □ |
| 1. Ich kann meine unterschiedlichen Gefühle anderen erklären. | □ | □ | □ | □ | □ | □ | □ |
| 1. Ich finde es nützlich, über meine Gefühle zu sprechen. | □ | □ | □ | □ | □ | □ | □ |
| 1. Ich kann mit anderen über die Veränderung meiner Gefühle sprechen. | □ | □ | □ | □ | □ | □ | □ |
| 1. Ich interessiere mich für die Gefühle anderer. | □ | □ | □ | □ | □ | □ | □ |
| 1. Ich kann widersprüchliche Gefühle bei anderen wahrnehmen. | □ | □ | □ | □ | □ | □ | □ |
| 1. Ich finde es bereichernd, Gefühle bei anderen zu erkennen. | □ | □ | □ | □ | □ | □ | □ |
| 1. Ich versuche Situationen durch die Augen des anderen zu sehen. | □ | □ | □ | □ | □ | □ | □ |
| 1. Ich finde es hilfreich, über die Gründe der Gefühle anderer nachzudenken. | □ | □ | □ | □ | □ | □ | □ |
| 1. Über die Zeit kann ich die Gefühle anderer besser verstehen. | □ | □ | □ | □ | □ | □ | □ |
| 1. Ich finde es spannend, darüber nachzudenken, woher die Gefühle anderer kommen. | □ | □ | □ | □ | □ | □ | □ |

Subskala Self: Item 1-5

Subskala Communicating: Item 6-9

Subskala Other: Item 10-16
