# Supplementary material for: Development and validation of the Mentalizing Emotions Questionnaire: A self-report measure for mentalizing emotions of the self and other
Source: PLoS One. 2024 May 6;19(5):e0300984. doi: 10.1371/journal.pone.0300984 (PMC11073734; doi:10.1371/journal.pone.0300984)
Supplement: S1 Table — (DOCX) [file pone.0300984.s004.docx]

Table I. 16-item MEQ divided into poles, elements and aspects.

| **Pole** | **Element** | **Aspect** | **Items** |
| --- | --- | --- | --- |
| Self | Identifying | *Interest* | I am interested in my emotions |
|  | Processing | *Interest* | I am interested in understanding my emotions. |
|  |  | *Multi-Perspective* | I try to understand the different reasons for my emotions. |
|  |  | *Acceptance* | I think it is helpful to understand the reasons of my emotions. |
|  |  | *Development-Perspective* | With some distance, I can understand my emotions in a new way. |
|  | Expressing | *Interest* | I find it exciting to talk about my emotions with others. |
|  |  | *Multi-Perspective* | I can explain my different emotions to others. |
|  |  | *Acceptance* | I think it is useful to talk about my emotions. |
|  |  | *Development-Perspective* | I can talk to others about how my emotions change. |
| Other | Identifying | *Interest* | I am interested in the emotions of others. |
|  |  | *Multi-Perspective* | I can perceive conflicting emotions in others. |
|  |  | *Acceptance* | I think it is enriching to recognize emotions in others. |
|  | Processing | *Interest* | I find it exciting to think about where others' emotions come from. |
|  |  | *Multi-Perspective* | I try to see situations through the other person's eyes. |
|  |  | *Acceptance* | I find it helpful to think about the reasons for others' emotions. |
|  |  | *Development-Perspective* | Over time, I can better understand the emotions of others. |
