# Supplementary material for: Development and validation of the Mentalizing Emotions Questionnaire: A self-report measure for mentalizing emotions of the self and other
Source: PLoS One. 2024 May 6;19(5):e0300984. doi: 10.1371/journal.pone.0300984 (PMC11073734; doi:10.1371/journal.pone.0300984)
Supplement: S2 Table — bold = items of the final MEQ. (DOCX) [file pone.0300984.s005.docx]

Table II. List of the original 23 items

| Item-Number |  |
| --- | --- |
| 1 | **I am interested in my emotions.** |
| 2 | I can perceive contradictory feelings within myself at the same time. |
| 3 | I accept my feelings as they are. |
| 4 | I can observe new feelings in myself from a distance. |
| 5 | **I am interested in understanding my emotions.** |
| 6 | **I try to understand the different reasons for my emotions.** |
| 7 | **I think it is helpful to understand the reasons of my emotions.** |
| 8 | **With some distance, I can understand my emotions in a new way.** |
| 9 | **I find it exciting to talk about my emotions with others.** |
| 10 | **I can explain my different emotions to others.** |
| 11 | **I think it is useful to talk about my emotions.** |
| 12 | **I can talk to others about how my emotions change.** |
| 13 | **I am interested in the emotions of others.** |
| 14 | **I can perceive conflicting emotions in others.** |
| 15 | **I think it is enriching to recognize emotions in others.** |
| 16 | I perceive the feelings of others differently over time. |
| 17 | **I try to see situations through the other person's eyes.** |
| 18 | **I find it helpful to think about the reasons for others' emotions.** |
| 19 | **Over time, I can better understand the emotions of others.** |
| 20 | **I find it exciting to think about where others' emotions come from.** |
| 21 | I talk to others about their conflicting feelings. |
| 22 | I find it good to talk to others about their feelings. |
| 23 | I find it important to tell others when I have developed a new understanding of their feelings. |

***bold*** *= items of the final MEQ*
